# Salidroside mitigates skeletal muscle atrophy in rats with cigarette smoke-induced COPD by up-regulating myogenin and down-regulating myostatin expression

**DOI:** 10.1042/BSR20190440

**Published:** 2019-11-22

**Authors:** Dan Zhang, Lihua Cao, Zhenshan Wang, Haoshen Feng, Xu Cai, Mingtao Xu, Menglu Li, Na Yu, Yan Yin, Wei Wang, Jian Kang

**Affiliations:** 1 Department of Respiratory Medicine, Institute of Respiratory Diseases, The First Affiliated Hospital of China Medical University, Shenyang 110001, Liaoning, China; 2 Department of Respiratory Medicine, The Second Affiliated Hospital of Dalian Medical University, Dalian 116000, Liaoning, China

**Keywords:** chronic obstructive pulmonary disease, Muscle dystrophy, Muscle-specific transcription factors, Salidroside

## Abstract

**Objectives**: The present study aimed at investigating the therapeutic effect of Salidroside on skeletal muscle atrophy in a rat model of cigarette smoking-induced chronic obstructive pulmonary disease (COPD) and its potential mechanisms.

**Methods**: Male Wistar rats were randomized, and treated intraperitoneally (IP) with vehicle (injectable water) or a low, medium or high dose of Salidroside, followed by exposure to cigarette smoking daily for 16 weeks. A healthy control received vehicle injection and air exposure. Their lung function, body weights and gastrocnemius (GN) weights, grip strength and cross-section area (CSA) of individual muscular fibers in the GN were measured. The levels of TNF-α, IL-6, malondialdehyde (MDA), superoxide dismutase (SOD), glutathione (GSH) in serum and GN tissues as well as myostatin and myogenin expression in GN tissues were measured.

**Results**: In comparison with that in the healthy control, long-term cigarette smoking induced emphysema, significantly impaired lung function, reduced body and GN weights and CSA values in rats, accompanied by significantly increased levels of TNF-α, IL-6 and MDA, but decreased levels of SOD and GSH in serum and GN tissues. Furthermore, cigarette smoking significantly up-regulated myostatin expression, but down-regulated myogenin expression in GN tissues. Salidroside treatment decreased emphysema, significantly ameliorated lung function, increased antioxidant, but reduced MDA, IL-6 and TNF-α levels in serum and GN tissues of rats, accompanied by decreased myostain, but increased myogenin expression in GN tissues.

**Conclusion:** Salidroside mitigates the long-term cigarette smoking-induced emphysema and skeletal muscle atrophy in rats by inhibiting oxidative stress and inflammatory responses and regulating muscle-specific transcription factor expression.

## Introduction

Chronic obstructive pulmonary disease (COPD) is a commonly chronic inflammatory disease and it will be the third most dead disease worldwide in 2030 [1]. Long-term heavy smoking is a risk factor for the development of COPD. This, together with air pollution, increases the incidence of COPD in smoking population. Many harmful components in cigarettes can damage epithelial cells in the respiratory tract and vascular endothelial cells, and cause chronic inflammation, leading to respiratory and cardiovascular diseases [2]. Furthermore, long-term heavy cigarette smoking and COPD can also result in skeletal muscular atrophy, accelerating the pathogenic process of COPD [3]. However, the pathogenesis of COPD-related skeletal muscle atrophy is unclear and there is no effective therapy for intervention of COPD-related skeletal muscle atrophy. Hence, understanding the pathogenesis and developing new therapies for the COPD-related skeletal muscle atrophy will be of significance in management of patients with COPD.

COPD can cause oxidative stress and chronic inflammation in the lung and skeletal muscles. The dysfunctional skeletal muscles also feedback deteriorate the lung damage and function, increasing morbidity and mortality of COPD patients [4,5]. Although malnutrition contributes to the process of skeletal muscle atrophy in COPD patients [6–8], whether other factors contribute to the development of COPD-related skeletal muscle atrophy have not been clarified. During the pathogenesis of COPD-related skeletal muscle atrophy, COPD-related hypoxia can cause inflammation, leading to production of high levels of pro-inflammatory cytokines in the muscular tissues, such as TNF-α and IL-6, which can further damage myocytes [9]. Furthermore, the myogenesis is positively regulated by muscle-specific transcription factors, such as myogenin, but inhibited by myostatin. The enhanced oxidative stress and inflammation can up-regulate myostain expression, but down-regulate myogenin expression, deteriorating the process of skeletal muscle atrophy. Therefore, therapeutic strategies to modulate these therapeutic targets may inhibit the process of COPD-related skeletal muscle atrophy.

Salidroside (*p*-hydroxyphenethyl-b-d-glucoside), is one of the major tyrosols of Chinese traditional herbs, and can be extracted from Rhodiolarosea L. Salidroside has been widely used as a traditional medicine in Eastern Europe and Asia. Previous studies have shown that salidroside has potent anti-oxidant [10], anti-inflammatory [11], anti-apoptotic [12], anti-depressive [13], anti-aging [14], neuroprotective [15,16], anti-cachexia [17], anti-COPD [18], cardioprotective activities [19,20], anti-fatigue and anoxia [21],and anti-Alzheimer’s disease properties [22,23]. However, little is known whether treatment with Salidroside can modulate the development and severity of COPD-related skeletal muscle atrophy.

The present study aimed at investigating the therapeutic effect of Salidroside treatment in a rat model of cigarette smoking-induced COPD-related skeletal muscle atrophy and its potential mechanisms.

## Materials and methods

### Animals

Male Wistar rats at 6–8 weeks of age and 160–180 g in body weight were from Liaoning Changsheng Biotechnology (Shenyang, China) and housed in a specific pathogen-free facility with free access to standard chow and water *ad libitum*. These rats were acclimated for 1 week prior to the start of the experiments and were housed in standard environmental conditions (25 ± 2°C and 12:12-h light:dark cycles). All experiments took place at Animal Facility of Department of Laboratory Animal Science in China Medical University. Animal experiments were carried out following the Institutional Animal Care and Use Committee, and were approved by the local Animal Experimentation Ethics Committee of China Medical University with number 2017111.

### Cigarette smoking and treatment

The rats were randomized and treated intraperitoneally (IP) with vehicle (sterile injectable water) or Salidroside (Shanghai Yuanye Bio-Technology, Shanghai, China) at 50 mg/kg (COPD+50 mg/kg group), 100 mg/kg (COPD+100 mg/kg group) or 200 mg/kg (COPD+200 mg/kg group), followed by cigarette smoking (Hongmei cigarettes, 13 mg CO, 13 mg Tar oil) for 30 min/day, twice per day at an interval of 6–8 h, 6 days per week for 16 weeks in an 8050™ inhalation exposure system (6–8% of smog concentration, Tianjin, China) [24–27]. A healthy control group of rats received vehicle injection and were exposed to normal air (*n*=8 per group).

### Lung function

The lung function of individual rats was measured by the ratios of forced expiratory volume (FEV) at 0.2 s (FEV_0.2_) to forced vital capacity (FVC) and peak expiratory flow (PEF) [28]. Briefly, individual rats were injected IP with 1% pentobarbital sodium (40 mg/kg) and maintained in an appropriate anesthesia. The trachea of each rat was cut, intubated, and attached to a ventilator (Res3020, Bestlab High-Tech, Beijing, China) at a respiratory rate of 75 beats/min with a tidal volume (5 ml/kg). The FEV_0.2_/FVC and PEF were measured using the AniRes2005 pulmonary mechanics analyzer (version 3.0, Bestlab High-Tech).

### Sample collection

Individual rats were subjected to chest surgery and their blood samples were obtained from the inferior vena cava. After coagulation, their sera were prepared by centrifugation. Their left lungs were perfused with 4% paraformaldehyde at a constant pressure of 25 cm H_2_O [27] and fixed in 4% paraformaldehyde for 48 h, followed by paraffin-embedded for histology and immunohistochemistry. Their right lungs were snap-frozen in liquid nitrogen. The gastrocnemius (GN) of each rat was dissected out. The lateral head and medial head of each GN were cut-fixed in 4% paraformaldehyde, and snap-frozen in liquid nitrogen, respectively.

### Histological analysis

The paraffin-embedded lung and the middle part of lateral head of GN tissue sections (4 µm) were stained with Hematoxylin and Eosin (HE) and photoimaged under a light microscope. The mean linear intercept (MLI) and mean alveolar number (MAN) of individual samples were measured [28,29]. Similarly, the cross-section area (CSA) of individual muscular fibers in the middle of the lateral head of GN was measured using the image proplus software (Media Cybernetics, Silver Spring, U.S.A.). A total of 27 fields selected randomly from three sections of each rat and three rats per group were measured.

### Assessment of grip strength, body and skeletal muscle weights

The total power of four limbs of each rat was measured for its grip power 1 day before anesthesia using an YLS-13A grip strength meter (Yiyan Technology Development, Shandong, China), according to the manufacturer’s instructions. The rats were food-fasted overnight and their body weights were measured before and 16 weeks after smoking. The body weight change was calculated. Their GN tissues were wet-weighed.

### Measurement of inflammatory cytokines and oxidant stress in serum and muscle homogenate samples

Individual GN tissues were homogenized and centrifuged, followed by quantifying the concentrations of proteins in their supernatants using bicinchoninic acid (BCA, Beyotime). The concentrations of TNF-α and IL-6 in individual sera and GN tissue homogenates were determined by enzyme-linked immunosorbent assay (ELISA, Cloud Clone, Wuhan, China), according to the manufacturer’s protocol. The levels of malondialdehyde (MDA), GSH and superoxide dismutase (SOD) inhibition ratio in individual sera and GN tissue homogenates were measured by chemical reactions using specific kits (Nanjing Jiancheng Bioengineering Institute, China), according to the manufacturer’s instructions. The SOD activity were calculated with this formula: SOD activity = SOD inhibition ratio × 24 × Sample dilution factor before test.

### Immunohistochemistry

The paraffin-embedded muscular tissue sections (5 μm) were deparaffinized, rehydrated and subjected to antigen retrieval in citrate buffer, pH 6.0 in a microwave for 20 min. The sections were treated with 3% H_2_O_2_ in methanol for 15 min and blocked with 10% goat sera. After being washed, the sections were incubated with anti-myostatin (1:400, Abcam), anti-myogenin (1:50, Santa Cruz Biotechnology, Santa Cruz, U.S.A.). The bound antibodies were detected using horseradish peroxidase (HRP)–conjugated secondary antibodies (Maixin, Fuzhou, China) and visualized with DAB, followed by photoimaging. The staining intensity of individual sections was evaluated using the Image-Pro Plus software (Media Cybernetics). The intensity of anti-myostatin and the percentages of nuclear anti-myogenin stained cells were measured in a blinded manner.

### Western blot analysis

The relative levels of myostain and myogenin to β-actin in individual muscle tissues were examined by Western blot. Briefly, the tissue homogenized samples (30 µg/lane) were resolved by sodium dodecyl sulfate/polyacrylamide gel electrophoresis (SDS/PAGE) and transferred on to polyvinylidene difluoride (PVDF) membranes (Millipore, Bedford, MA, U.S.A.). The membranes were blocked with 5% fat-free dry milk in TBST (Tris-Buffered Saline Tween-20) and incubated with anti-myostatin (1:500, Abcam), anti-myogenin (1:1000, Santa Cruz Biotechnology), anti-β-actin (1:1000, Cloud Clone). The bound antibodies were detected with HRP–conjugated secondary antibodies and visualized using an enhanced chemiluminescent reagents (Thermo). The data were analyzed using the ImageJ software.

### RNA extraction and quantitative real-time PCR

Total RNA was extracted from individual GN tissue samples and reverse transcribed into cDNA using a reverse transcription kit (Takara, Japan). The relative levels of myostatin and myogenin to control β-actin mRNA transcripts in individual muscle samples were assessed by quantitative RT-PCR using SYBR Premix Ex Taq™ (Takara, Japan) and specific primers in LightCycler 480 (Roche, Switzerland). The sequences of primers were forward 5′-ATTATCACGCTACCACGGAAACA-3′ and reverse 5′-AGCTGGGCCTTTACCACTTTG-3′ for myostatin; forward 5′-TGCACATCTGTTCGACTCTCTTC-3′ and reverse 5′-CCCTATCGTTCCCTCCCTTC-3′ for myogenin; forward 5′-TGTCACCAACTGGGACGATA-3′ and reverse 5′-GGGGTGTTGAAGGTCTCAAA-3′ for β-actin. The data were normalized to β-actin and analyzed by 2^−ΔΔ*C*_t_^.

### Statistical analysis

Data are present as the means ± standard deviation (SD). The difference among the groups was analyzed by one-way ANOVA and post hoc Bonferroni’s test using the GraphPad Prism 5.0 software (San Diego, CA, U.S.A.). A *P*-value of less than 0.05 was considered statistically significant.

## Results

### Salidroside treatment ameliorates lung function in rats with cigarette smoking-induced COPD

**Figure 1:**
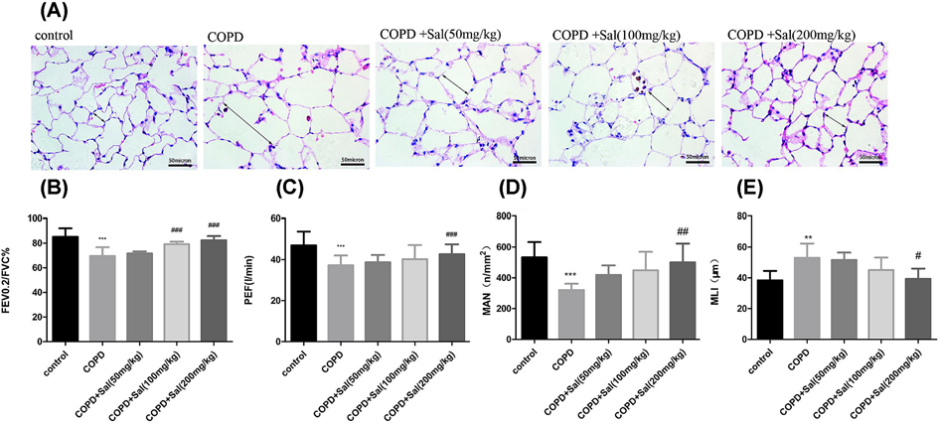
Salidroside alleviates emphysema and lung function in rats with cigarette smoking-induced COPD (**A**) Histological changes in lung tissues. Scale bars = 50 μm. (**B,C**) Lung functional measurements. (**D,E**) Lung histological measurements. Data are representative images (magnification ×400) or the mean ± SD of each group (*n*=8 rats per group). **P<0.01, ***P<0.001 vs. the control group, ^#^*P*<0.05, ^##^*P*<0.01, ^###^*P*<0.001 vs. the COPD group.

Long-term heavy cigarette smoking is the highest risk factor for the development of COPD, which usually affects the bronchioles and alveoli, leading to emphysema. To determine the therapeutic effect of Salidroside, Wistar rats were subjected to cigarette smoking, randomized and treated with vehicle or different doses of Salidroside daily for 16 weeks. Histological examination indicated that compared with the healthy control, there was obvious emphysema and enlarged alveoli in the lung tissues of the COPD group of rats while Salidroside treatment mitigated and abrogated in the COPD-related emphysema and alveolus enlargement in the lungs of rats in a dose-dependent manner (Figure 1A). Further analyses indicated that the ratios of FEV_0.2_/FVC and the values of PEF and MAN were significantly reduced in the COPD group of rats (*P*<0.001) while Salidroside treatment at a medium or high dose significantly mitigated the COPD-decreased ratios of FEV_0.2_/FVC in the rats (Figure 1B–D). Similarly, Salidroside treatment at a high dose also significantly improved the values of PEF and MAN in the rats, compared with that in the COPD group (*P*<0.001, *P*<0.01, Figure 1C,D). Moreover, while the values of MLI in the COPD group were significantly higher than the healthy control (*P*<0.01), the values of MLI in the rats that had been treated with a high dose of Salidroside were significantly reduced (*P*<0.05, Figure 1E). Hence, Salidroside treatment significantly mitigated the heavy smoking-related lung damage and ameliorated the lung function in rats with COPD.

### Salidroside treatment mitigates the COPD-mediated skeletal muscle atrophy in rats

**Figure 2:**
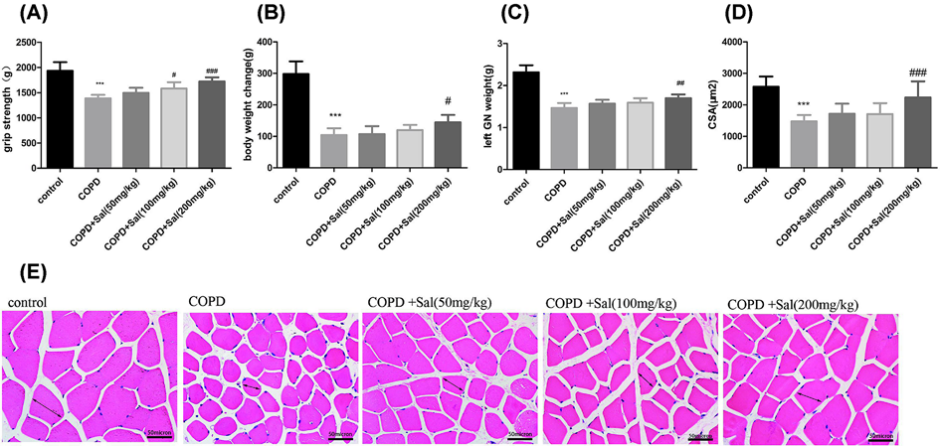
Salidroside treatment mitigates the COPD-mediated skeletal muscle atrophy in rats Rats were subjected to air or cigarette smoking and treated with the different doses of Salidroside daily for 16 weeks, and their grip strengths (**A**) and body weights (**B**) were measured. The left GN weights (**C**) and CSAs (**D,E**) were measured. Scale bars = 50 μm. Data are representative images (magnification ×400) or the mean ± SD of each group (*n*=8 rats per group). ***P<0.001 vs. the control group, ^#^*P*<0.05, ^##^*P*<0.01, ^###^*P*<0.001 vs. the COPD group.

Long-term smoking-induced COPD can cause skeletal muscle atrophy, reduce skeletal muscle weights, and change body weight and grip strength [30]. To determine the consequence of COPD and Salidroside treatment, the grip strengths of individual groups of rats were measured. The grip strengths in the rats that had been treated with a medium or high dose of Salidroside were significantly higher than that in the COPD group, but remained lower than that in the healthy group (*P*<0.05, *P*<0.001, Figure 2A). Similarly, the body and GN weights in the rats received a high dose of Salidroside were significantly greater than that in the COPD group, but were less than that in the healthy group (*P*<0.05, *P*<0.01, Figure 2B,C). Furthermore, histological examination and quantitative analyses revealed that the CSA values in the rats received a high dose of Salidroside were significantly larger than that in the COPD group (*P*<0.001 for both, Figure 2D,E). Thus, Salidroside treatment significantly mitigated the COPD-mediated skeletal muscle atrophy in rats.

### Salidroside treatment mitigates the oxidative stress-related pro-inflammatory cytokine production in rats

**Figure 3:**
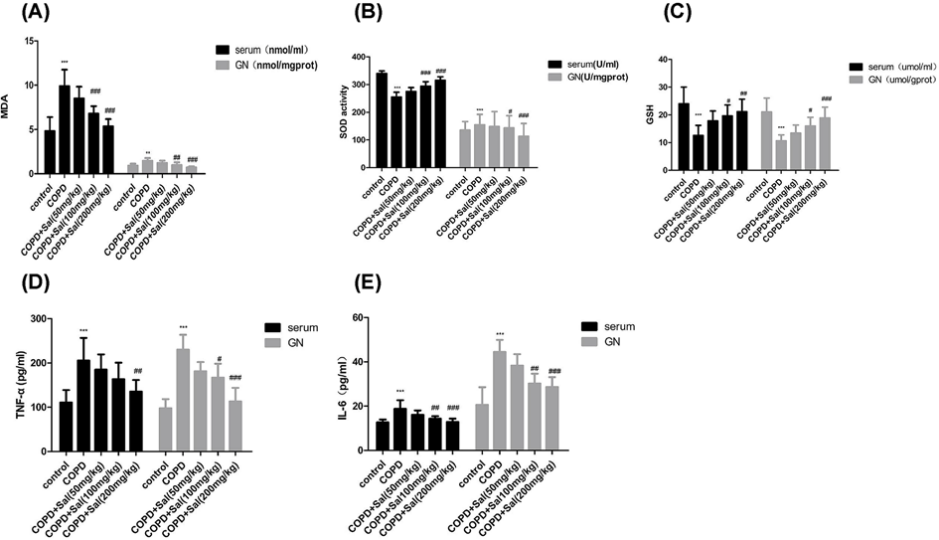
Salidroside treatment reduces the oxidative stress-related pro-inflammatory cytokine production in rats (**A–C**) The levels of MDA, SOD and GSH in serum and GN tissue homogenates of individual rats were measured. (**D,E**) The levels of TNT-α and IL-6 in serum and GN tissue homogenates of individual rats. Data are presented as the mean ± SD of each group (*n*=8 rats per group) from three independent assays. ***P<0.001 vs. the control group, ^#^*P*<0.05, ^##^*P*<0.01, ^###^*P*<0.001 vs. the COPD group.

Long-term cigarette smoking can induce oxidative stress, which induces pro-inflammatory cytokine production in the skeletal muscles. To understand the pathogenesis of the COPD-related skeletal muscle atrophy, the levels of MDA, SOD and GSH in serum and GN tissues of individual rats were measured. The levels of MDA in serum and GN tissues of the rats that had been treated with Salidroside at a medium or high dose were similar to that of the healthy control, but significantly lower than that of the COPD group (*P*<0.01, *P*<0.001, Figure 3A). In contrast, the levels of SOD and GSH in serum and GN tissues of the rats that had been treated with Salidroside at a medium or high dose were similar to that of the healthy control, but were significantly higher than that in the COPD group (Figure 3B,C). Further analysis indicated that the levels of TNF-α and IL-6 in serum and GN tissues of the rats that had been treated with Salidroside at a medium or high dose were comparable with that in the healthy group, but were significantly lower than that in the COPD group (*P*<0.01, *P*<0.001, Figure 3D,E). Collectively, such data demonstrated that Salidroside treatment at a medium or higher dose significantly mitigated the COPD-related oxidative stress and inflammatory cytokine production in rats.

### Salidroside treatment alters the myostatin and myogenin expression in GN tissues of rats with COPD

**Figure 4:**
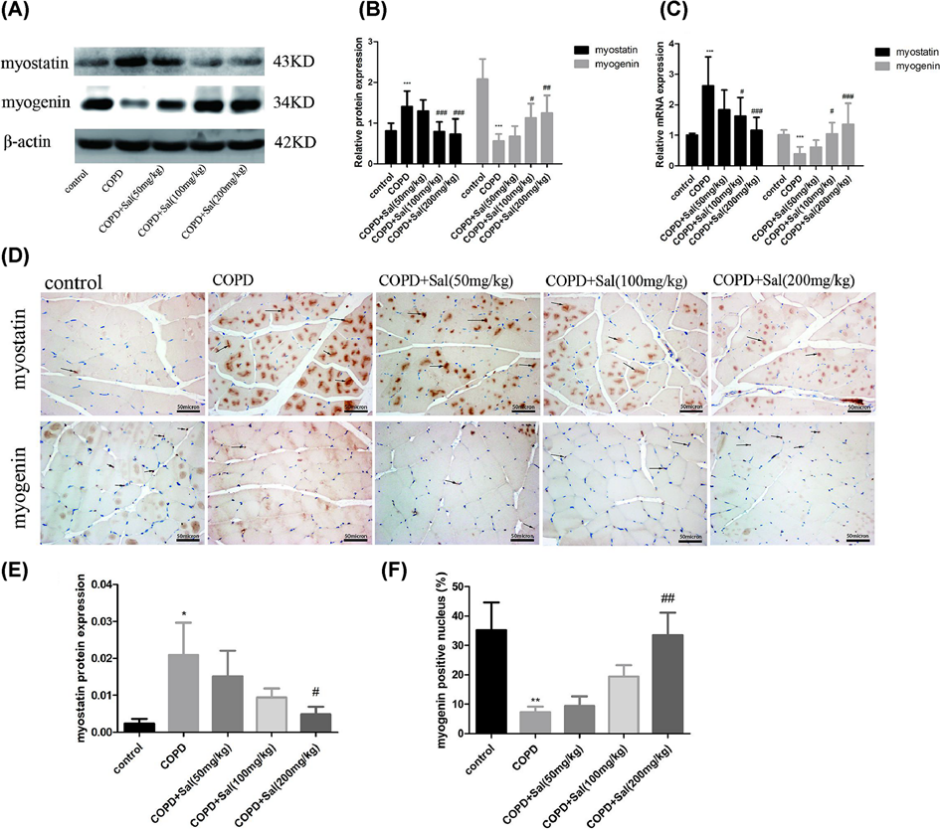
Salidrosides reduce the myostatin expression and enhance the myogenin expression in GN tissues of rats (**A,B**) Western blot analysis of the relative levels of myostatin and myogenin to β-actin expression in the GN tissues of individual groups of rats. (**C**) Quantitative RT-PCR analysis for the relative levels of myostatin and myogenin mRNA transcripts in the GN tissues of individual rats. (**D–F**) Immunohistochemistry analysis of myostatin expression and the percentages of nuclear myogenin cells in the GN tissues of individual rats. Scale bars = 50 μm. Data are representative images (magnification ×400) or present as the mean ± SD of each group (*n*=8) from three separate experiments. *P<0.05, **P<0.01, ***P<0.001 vs. the control group, ^#^*P*<0.05, ^##^*P*<0.01, ^###^*P*<0.001, vs. the COPD group.

It is well known that myostatin can inhibit myogenesis while myogenin is a muscular transcription factor to promote myogenesis [31–34]. To further understand the action of Salidroside treatment in regulating the COPD-related skeletal muscle atrophy, the relative levels of myostatin and myogenin expression in the GN tissue samples were determined by Western blot (Figure 4A). Quantitative analyses revealed that the relative levels of myostatin in the GN tissues from the rats that had been treated with Salidroside at a medium or high dose were comparable with that in the healthy group, but significantly lower than that in the COPD group (*P*<0.001 for all, Figure 4B). In contrast, the relative levels of myogenin expression in GN tissues from the rats received a medium or high dose of Salidroside were significantly higher than that in the COPD group (*P*<0.05, *P*<0.01, Figure 4B). Similar patterns of myostain and myogenin mRNA transcripts and protein expression were detected by quantitative RT-PCR and immunohistochemistry in different groups of rats (Figure 4C–F). Therefore, Salidroside treatment significantly decreased myostatin expression, but increased myogenin expression in the GN tissues of COPD rats, contributing to protection from the COPD-induced skeletal muscle atrophy in rats.

## Discussion

Previous studies have shown that long-term cigarette smoking can cause COPD, which is characterized by emphysema and impaired lung function [29,35]. In this study, we found that cigarette smoking for 16 weeks decreased the ratios of FEV_0.2_/FVC, the values of PEF and MAN, but increased MLI values, consistent with characteristics of emphysema and COPD [28]. Furthermore, long-term cigarette smoking significantly decreased body and GN weights by reducing CSA of muscular fibers in the GN and functionally imparing grip strengths in rats, hallmarks of COPD-related skeletal muscle atrophy. Conceivably, the skeletal muscle atrophy also occurred in other muscles, particularly in the diaphragm and quadriceps muscles, leading to its fatigue, contributing to poor quality of life and respiratory failure [4,36]. More importantly, we demonstrated that Salidroside treatment, particularly with a high dose, significantly mitigated and abrogated the cigarette smoking-induced emphysema and COPD-impaired lung function and decreased the COPD-related skeletal muscle atrophy in rats in a dose-dependent manner. Such novel data suggest that Salidroside may be a promising candidate for design of new therapies for intervention of smoking-related COPD.

Long-term cigarette smoking can cause oxidative stress and inflammation, contributing to skeletal muscle atrophy [37]. In this study, we found that while cigarette smoking significantly increased the levels of MDA, TNF-α and IL-6, but decreased levels of SOD and GSH in serum and GN tissues of rats. Salidroside treatment significantly mitigated smoking-related oxidative stress and pro-inflammatory cytokine production in rats, consistent with its antioxidant and anti-inflammatory activity, which may contribute to its therapeutic effect on inhibiting the COPD-related skeletal muscle atrophy [10,11,18]. Given that Salidroside has been demonstrated to be relatively safe for humans Salidroside may be valuable for intervention of other inflammatory diseases [38].

Myogenic regulatory factors (MRFs) in the MyoD family are mainly muscle-specific transcription factors, including MyoD, myogenin, myf-5 and MRF4, and are crucial for regulating myogenesis [31,32]. Myogenin is expressed in all skeletal muscles and is a key factor to promote the terminal differentiation of myocytes. MRFs can promote muscle damage repair in animal models of trauma [39,40], neurogenic and myogenic myopathy [41]. On the other hand, myostatin has structure similar to TGF-β and can inhibit the myogenesis [33,34]. Myostatin^−/−^ mice display increased body weights and larger CSA of muscular fibers [34]. In this study, we found that long-term cigarette smoking significantly increased levels of myostatin expression, but decreased myogenin expression in the GN tissues of rats. Treatment with Salidroside at a medium or high dose completely abrogated the cigarette smoking up-regulated myostatin expression and significantly mitigated the COPD-decreased myogenin expression in GN tissues of rats. These data extended previous findings on the pathogenic role of myostatin in tumors [17,42–44], sarcopenia [45], neuromuscular diseases [46,47] and COPD [48–50], and support the notion that myostatin participates in the pathogenic process of skeletal muscle damage. Actually, recent studies reveal a three-fold increase in myostatin mRNA transcripts in the vastus lateralis muscles in COPD patients with significant quadriceps weakness [51,52]. Furthermore, higher plasma myostatin levels are detected in COPD patients with corpulmonale complication [53,54]. It is possible that Salidroside and its metabolites may inhibit oxidative stress, inflammation and myostatin expression in COPD rats. These, together with promoting myogenin expression, promote the repair of damaged muscles to inhibit the COPD-related skeletal muscle atrophy. We are interested in further investigating the molecular mechanisms underlying the action of Salidroside in regulating the expression of these molecules during the development and progression of COPD.

In conclusion, our data indicated that Salidroside treatment significantly reduced the cigarette smoking-induced emphysema and ameliorated lung function in rats. Furthermore, Salidroside treatment significantly mitigated the COPD-induced skeletal muscle atrophy by reducing oxidative stress and the production of pro-inflammatory cytokines in serum and the GN tissues and altering myostatin and myogenin expression in the GN tissues. Therefore, our findings may provide a basis for design of new therapies for COPD-related skeletal muscle atrophy.

## Abbreviations

COPD: chronic obstructive pulmonary disease
CSA: cross-section area
FEV: forced expiratory volume
FEV_0.2_: forced expiratory volume at 0.2 s
FVC: forced vital capacity
GN: gastrocnemius
GSH: glutathione
HRP: horseradish peroxidase
IP: intraperitoneally
MAN: mean alveolar number
MDA: malondialdehyde
MLI: mean linear intercept
MRF: myogenic regulatory factor
PEF: peak expiratory flow
Salidroside: *p*-hydroxyphenethyl-b-d-glucoside
SOD: superoxide dismutase
TBST: Tris-Buffered Saline Tween-20
